# Feature Selection for the Automated Detection of Metaphase Chromosomes: Performance Comparison Using a Receiver Operating Characteristic Method

**DOI:** 10.1155/2014/565392

**Published:** 2014-11-11

**Authors:** Yuchen Qiu, Jie Song, Xianglan Lu, Yuhua Li, Bin Zheng, Shibo Li, Hong Liu

**Affiliations:** ^1^Center for Bioengineering and School of Electrical and Computer Engineering, University of Oklahoma, 101 David L. Boren Boulevard, Norman, OK 73019, USA; ^2^Department of Biology, Mudanjiang Medical University, Mudanjiang 157011, China; ^3^Department of Pediatrics, University of Oklahoma Health Sciences Center, Oklahoma City, OK 73104, USA

## Abstract

*Background*. The purpose of this study is to identify a set of features for optimizing the performance of metaphase chromosome detection under high throughput scanning microscopy. In the development of computer-aided detection (CAD) scheme, feature selection is critically important, as it directly determines the accuracy of the scheme. Although many features have been examined previously, selecting optimal features is often application oriented. *Methods*. In this experiment, 200 bone marrow cells were first acquired by a high throughput scanning microscope. Then 9 different features were applied individually to group captured images into the clinically analyzable and unanalyzable classes. The performance of these different methods was assessed by a receiving operating characteristic (ROC) method. *Results*. The results show that using the number of labeled regions on each acquired image is suitable for the first on-line CAD scheme. For the second off-line CAD scheme, it would be suggested to combine four feature extraction methods including the number of labeled regions, average regions area, average region pixel value, and the standard deviation of either region distance or circularity. *Conclusion*. This study demonstrates an effective method of feature selection and comparison to facilitate the optimization of the CAD schemes for high throughput scanning microscope in the future.

## 1. Introduction

Chromosome imaging and karyotyping is an important and widely used clinical method for the diagnosis of genetic related diseases and cancers [1–3]. For this technique, identifying a sufficiently large number of pathologically analyzable metaphase chromosomes is critically important for the final accuracy of cancer diagnosis and residual cancer cell detection. At present, the chromosome identification is a two-step semiautomatic procedure [4]. Commercialized automatic scanners first scan and locate the clinically useful cells under low magnification state (i.e., 10x objective lens). Second, clinicians have to manually move back to these detected locations again for high resolution image acquisition (i.e., under 100x objective lens), which is labor intensive and time consuming. In addition, it also creates substantial interobserver variation due to the bias of cell selection (i.e., the tendency towards selecting cells with good morphology). Therefore, the automatic scanning techniques are proposed and developed in the last 20 years, in an attempt to reduce the clinicians' workload and improve the diagnostic accuracy and consistency [5].

Recently, a new high throughput scanning method is reported in our laboratory [6]. Comparing to the currently commercialized scanners, our new method can accomplish a one-step scanning procedure, which directly provides the high resolution chromosome images for the following diagnosis (i.e., under 100x objective lens) [6–8]. In order to apply this technique to the future practice, on-line and off-line computer-aided detection (CAD) schemes are needed to be integrated into the image scanning procedure, for selecting the clinically analyzable metaphase chromosomes [6]. The on-line CAD scheme synchronizes with the high speed image scanning process and initially detects the analyzable cells, while the off-line CAD scheme is applied after scanning, to further select the analyzable images on the results firstly processed by the on-line scheme.

In order to determine whether the image contains analyzable chromosomes, both on-line and off-line CAD schemes extract and compute a set of image features from the segmented region of interest (ROI) on the acquired image. Therefore, selecting optimal and robust feature set will directly determine the final accuracy of the entire scheme, which is critically important for the CAD scheme. In the last several years, investigating new features has received extensive research interest and a series of different methods have been reported [9–13]. However, the effectiveness of feature selection is often task dependent or application oriented. It is difficult to directly compare these previously published chromosome features for our CAD scheme, as these methods are applied under different scanning conditions and evaluated using different standards. Therefore, it is necessary to investigate how to effectively evaluate these features under the high throughput scanning condition.

For this purpose, we performed a new study in which a certain number of bone marrow chromosomes were scanned and imaged under the high throughput scanning prototype. Different image features were computed by our CAD scheme to detect and classify the analyzable cells among the scanned images. The performance of the features was assessed and compared using a receiver operating characteristic (ROC) data analysis method. The detailed experimental methods and results are reported as follows.

## 2. Materials and Methods

During the specimen slide scanning, only a small number of scanned images are qualified for the clinical examination, as most of the scanned image regions contain unanalyzable cells due to the sample processing in the genetic laboratory. Therefore, a CAD scheme is applied to detect and identify the image regions of interest (ROIs) depicting the analyzable chromosomes. To develop an effective and robust CAD scheme, feature extraction is a critically important step in the CAD development and optimization [5, 9, 10, 13].

In this investigation, different features were assessed under a high throughput scanner. The entire assessment includes the following three steps. First, a number of 200 cells were randomly selected from bone marrow specimens. All these selected cells were obtained as image ROI, using our recently developed scanning microscopy prototype [6]. The size of each ROI is 3488 × 2048 pixels, which is sufficient to cover the region of the entire cluster of metaphase chromosomes. Each cell was captured under a 100x objective lens, by a time delay integration (TDI) camera with a pixel size of 7 *μ*m.

Second, the CAD scheme computed a number of image features for the captured ROI. In the last twenty years, many features have been investigated for the chromosome identification, which include morphology parameters such as size or circularity [10], centromeric index [14–16], relative length [14–16], density profile of the band pattern [15–17], and wavelet based multiresolution curvature [18]. However, except for the morphology parameters, all the other features (centromeric index, relative length, etc.) are developed to distinguish the detailed difference between the various chromosome band patterns for the karyotyping. Thus, these karyotyping features require more complicated image segmentation to separate the overlapped band patterns [5, 19, 20] and also have high computing complexity, which are not necessary and may reduce the efficiency of the CAD scheme. Comparatively, the morphology features can balance the efficiency and the effectiveness for our application. Therefore, in this study, the feature pool includes a number of nine different morphology features, which are the most representative for the metaphase chromosome classifications [9, 10, 13]. They are detailed as follows.

1. The number of labeled regions [10]: after applying the region growth and labeling algorithm, the CAD detects and counts the number of isolated “chromosomes.”
2. Average region pixel intensity [21]: the CAD computes the average pixel intensity value for all the labeled “chromosomes” on the image.
3. Standard deviation (STD) of the region pixel intensity [21]: the CAD first computes the average pixel intensity for each labeled region and then calculates the standard deviation of the region pixel intensity for all the labeled “chromosomes.”
4. Average region area [21]: the CAD computes the area of each labeled region (“chromosome”) by counting the number of pixels contained in the region. The average region area for the entire image was computed by averaging the region area of all the labeled regions.
5. STD of the region area [21]: the CAD computes the standard deviation of the region area for all the labeled regions contained on the entire image.
6. Average region circularity [10, 22]: in order to calculate this feature, the circularity of each labeled region was first computed. For each region, an equivalent circle was created, and this circle has the same area as the labeled region. The CAD then computes the overlapped area (*A*_*o*_) between the equivalent circle and the entire region. The region circularity is then defined as the ratio between the overlapped area (*A*_*o*_) and entire regions area (*A*): *A*_*o*_/*A*. After that, the circularities of all the regions were averaged for the entire image.
7. STD of the region circularity [10, 22]: the CAD computed the standard deviation of the circularities of all the labeled regions within the entire image.
8. The average region distance [10]: the CAD first computes the global gravity center (*x*_*g*_, *y*_*g*_) of all the labeled regions. The radial distance is then defined as the distance between the gravity center (*x*, *y*) of each labeled region and global gravity center (*x*_*g*_, *y*_*g*_). The radial distances of all the regions were averaged as the average region distance.
9. STD of the region distance [10]: the CAD computes the standard deviation of the region distances for all the labeled regions on the image.

Third, the performance of the CAD scheme was objectively assessed. Currently, in most of the previous studies, the performance is evaluated by CAD-observer agreement coefficient (Kappa coefficient) [23] or the classification accuracy [24, 25]. The Kappa coefficient is still a subjective evaluation, as it cannot avoid the interobserver variance. The classification accuracy only reveals the feature performance (i.e., true positive fraction (TPF) and false positive fractions (FPF)) at one previously determined threshold. However, in the future clinical application, the tested features might be applied at other possible thresholds. Thus, the receiving operating characteristic (ROC) method [26–28] is utilized in this investigation, which reflects the trade-off between the TPF and FPF at various thresholds. For each feature, a ROC curve was computed by estimating the true positive fraction (TPF) at different false positive fractions (FPF), which are determined based on the discrimination threshold [26]. The cell is considered as clinically analyzable (positive) or unanalyzable (negative) if the feature is within or outside the discrimination threshold, respectively. In the realistic application, the distribution of the true and false positive cases can be approximated as normal distributions [26, 28]. In order to estimate the TPF at different FPF, the data were categorized by several discrimination thresholds. At each threshold, the TPF and FPF were estimated. The ROC curves were estimated by maximum likelihood method, using the ROCKIT program [26].

In this investigation, the area under the curve (AUC) was first computed [26]. The features with an AUC under or close to 0.5 were discarded, as their performances are not better than the random decision. Then, each pair of the remained features was compared and the difference significance among these feature classifying performances was determined by the partially paired model [29, 30]. The feature correlation was also calculated to analyze the statistical independence of the features. Since all the selected features have low computing complexity, the executing time will not seriously affect the efficiency of the CAD scheme. The executing time was not explicitly assessed in the investigation.

In the high throughput scanning microscopy, the on-line and off-line CAD schemes have different requirements [6]. The on-line CAD scheme has to synchronize with the high speed image scanning; thus only the features with best performance will be selected, to reduce the algorithm complexity and improve the efficiency. The off-line CAD scheme, however, will select the final results for the diagnosis. Therefore, we should combine the advantages of all the tested meaningful features, to select the analyzable cells among the on-line results.

## 3. Results

**Figure 1:**
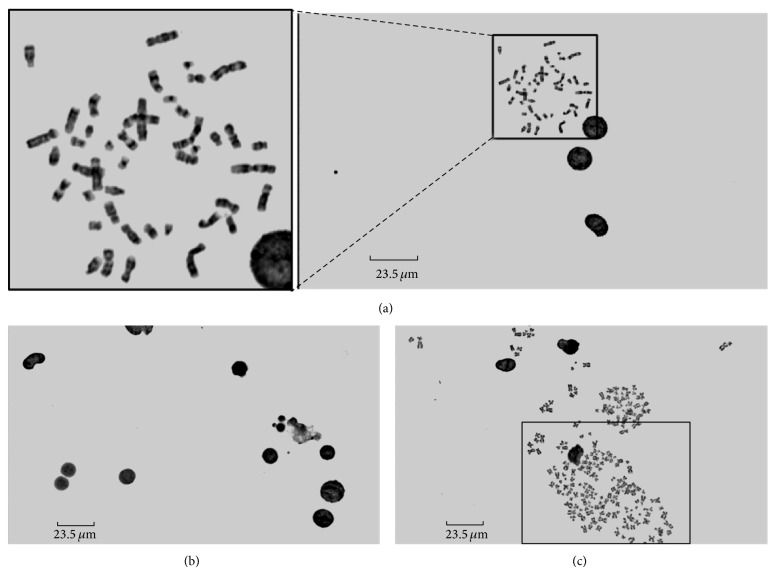
Examples of the microscopic images captured by the high throughput scanner. The cells were acquried under 100x objective lens and imaged by a TDI detector with a pixel size of 7 *μ*m. Image (a) contains a clinically analyzable region of interest (ROI). Images (b) and (c) contain interphase cells and overlapped metaphase chromosomes, respectively, which are unanalyzable for the diagnosis.

Figure 1 shows three images acquired by the high throughput scanner.  Figure 1(a) contains a clinically analyzable region of interest (ROI), while Figures 1(b) and 1(c) do not contain analyzable chromosomes for diagnostic purpose.  Figure 1(b) only contains interphase cells.  Figure 1(c) has more than one metaphase cells, and they are overlapped with each other. It can be seen that all the metaphase chromosomes are located in a certain area of the image. Comparing to the interphase cells, the metaphase chromosome is bright and has small size. In addition, the shape of the metaphase chromosome is totally different from the approximately circular interphase cells. The number of labeled regions in (a) is much larger than (b), as a normal human cell contains 46 chromosomes and one unanalyzable image would not contain so many interphase cells. For some unanalyzable images like (c), it has more than one metaphase cells, so the number of labeled regions is much larger than (a).

**Figure 2:**
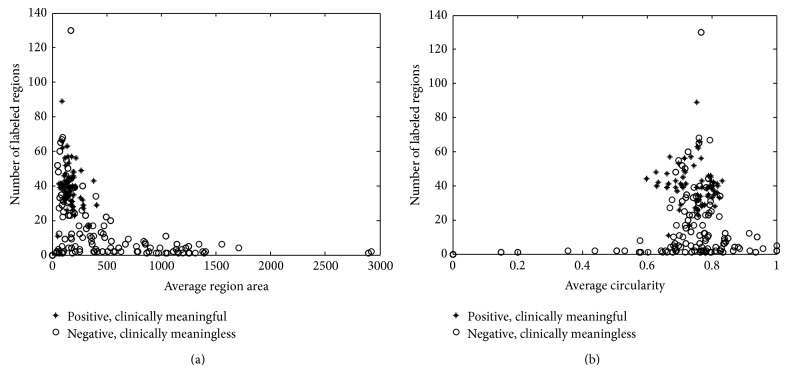
The feature scatter diagram of dataset. It contains 67 clinically meaningful and 133 clinical unanalyzable chromosomes. The vertical axis reflects the number of labeled regions, while the horizontal axis represents (a) average region area, (b) average region circularity.

Figure 2 shows two scatter diagrams of the dataset demonstrating the relationship of the feature distribution between analyzable and unanalyzable ROIs.  Figure 2(a) is a scatter diagram between average region area and the number of labeled regions. Since most of the chromosomes can be labeled as individual region, most of the analyzable cells have more labeled regions. In addition, the metaphase chromosomes are much smaller than the unanalyzable interphase cells. Thus most of the clinically analyzable cells are located in the up left corner of the diagram. Some unanalyzable cells are also located in the up left corner, because some unanalyzable cells contain many metaphase chromosomes, as illustrated in Figure 1(c).  Figure 2(b) is the feature distribution between the number of labeled regions and the average region circularity. It can be seen that a lot of features are overlapped in the horizontal direction, as some short analyzable chromosomes also have a large circularity and the captured analyzable images also contain interphase cells with large circularity.

**Figure 3:**
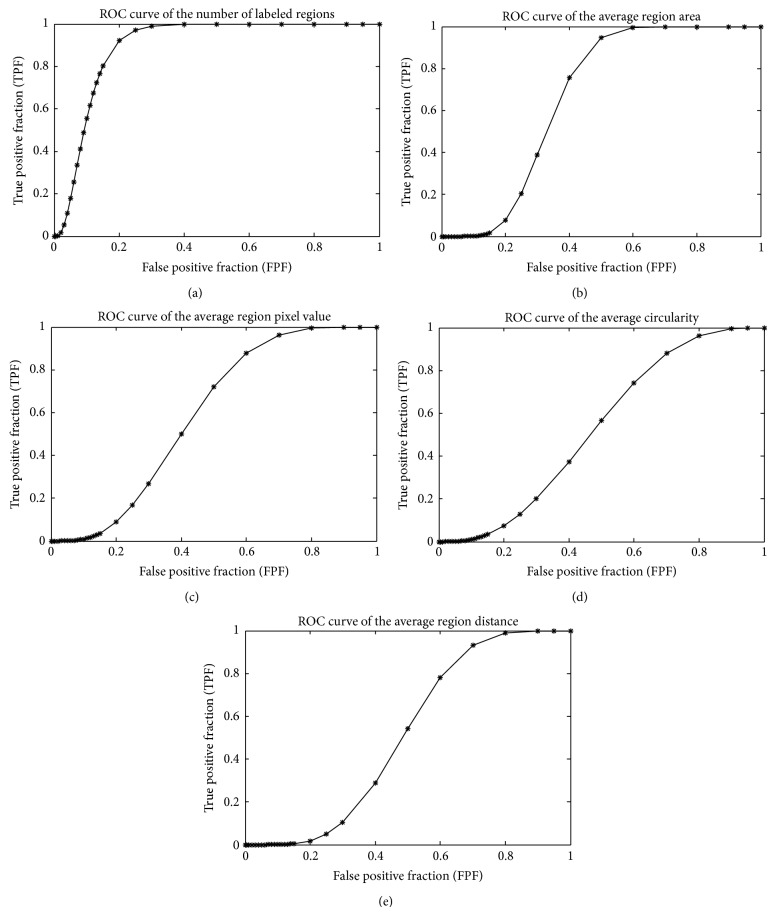
The estimated ROC curve for different extracted features: (a) the number of labeled regions, (b) average region area, (c) average region pixel intensity, (d) average region circularity, and (e) average region distance. Accordingly, the calculated area under curve (AUC) is (a) 0.896 ± 0.023, (b) 0.666 ± 0.037, (c) 0.592 ± 0.039, (d) 0.531 ± 0.040, and (e) 0.516 ± 0.039, respectively.

**Figure 4:**
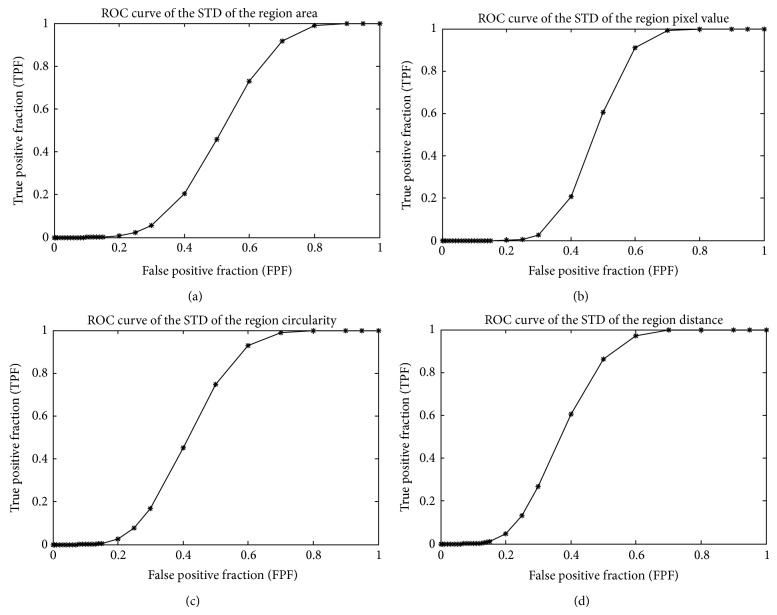
The ROC of the standard deviation of different features including (a) region area, (b) region pixel intensity, (c) region circularity, and (d) region distance. The AUC of the ROC curves is (a) 0.486 ± 0.039, (b) 0.524 ± 0.039, (c) 0.581 ± 0.039, and (d) 0.625 ± 0.038.

Figures 3 and 4 demonstrate and compare a set of ROC curves computed from different features. Among all these features, the number of labeled regions, average region area, average region pixel value, STD of the region circularity, and STD of the region distance demonstrate high discriminatory ability, as the area under curve (AUC) of the other four features is under or very close to 0.5. Among these features, the AUC of the number of labeled regions is 0.896 ± 0.023, which is significantly better than the AUC of the average region area (0.666 ± 0.037), average region pixel intesity (0.592 ± 0.039), STD of the circularity (0.581 ± 0.039), and STD of the region distance (0.625 ± 0.038). Although the AUC of the other four features ranges from 0.666 to 0.581, the differences between these features are not statistically significant (*P* ≥ 0.05), as illustrated in Table 1.

Table 2 shows the correlation coefficient between each pair of the investigated features. It can be seen that the number of labeled regions, average region area, and average region pixel value are relatively independent features, as the correlation coefficient between these features is smaller than 0.5. The STD of the region circularity and the STD of the region distance are related to each other, but each of these two features is also independent of the other three features (the number of labeled regions, average region area, and average region pixel value).

For the high throughput scanning, both the on-line and off-line CAD schemes are applied [6]. The on-line scheme is synchronized with the high speed image scanning to initially detect the analyzable cells. Thus it requires high efficiency and high sensitivity, which occurs with the cost of low specificity (high false positive fraction). Since the on-line results contain many images depicting unanalyzable chromosomes, the off-line CAD scheme requires both high sensitivity (false positive fraction) and specificity (false positive fraction), to finally select the analyzable images while discarding the others. Among all the five meaningful features, the number of labeled regions has better performance than the others; thus it is suggested as the only feature for the on-line CAD scheme, to satisfy the real time requirement. After the on-line processing, a number of 1000–3000 ROIs are saved [6], among which only 10–30 ROIs contain analyzable metaphase cells for the following diagnosis. Thus the off-line CAD scheme requires high specificity to discard most of the false positive images selected by the on-line CAD scheme. Furthermore, using the modern classifiers, the CAD scheme is able to combine more than one extracted feature, to achieve a better accuracy [31–34]. As mentioned before, it is not necessary to apply both the STD of the region distance and circularity because they are correlated features. Therefore, it is recommended to combine the number of labeled regions, average region area, average region pixel value, and standard deviation of either region distance or circularity, for the off-line CAD schemes.

**Figure 5:**
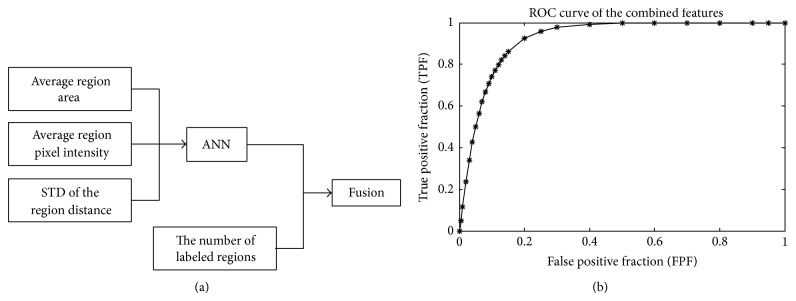
(a) The architecture of the classifier for the four selected features selected in the off-line CAD scheme. (b) The estimated ROC curve of the classifier. The AUC of the ROC curve is 0.9244 ± 0.026.

In order to verify the performance of the selected features for the off-line CAD scheme, a two-level classifier was designed, as demonstrated in Figure 5(a). In the first level, an ANN classifier combines the average region area, average pixel intensity, and the STD of the region distance to provide a score ranging from 0 to 1 (0 is definitely negative and 1 is definitely positive). The ANN score is fused with the normalized value of the number of labeled regions in the second level, by the minimum rule [35]. The estimated ROC curve of the classifier is shown in Figure 5(b). The AUC is 0.924 ± 0.026, which is superior to any individual feature in this combination.

## 4. Discussion

High throughput scanning microscopy is a promising method to digitalize the cells depicted on the clinical slides. Comparing to the commercialized scanners, this new prototype can scan and acquire the high resolution chromosome images directly for the diagnosis. Since only a small number of cells contained on the slide are actually analyzable for the diagnosis, a CAD scheme is needed to select the ROIs depicting analyzable chromosomes for the following diagnosis. For the development of a robust CAD schemes, the feature selection is critically important, which may directly determine the final performance of the CAD scheme. Thus the CAD designers need to carefully select the most suitable features, to satisfy the different requirements of the various CAD schemes.

In the last several years, many feature extraction methods are reported, which can effectively identify the pathologically analyzable metaphase chromosomes [9–13]. However, it is difficult to compare the reported results directly, as these features were applied on the different datasets and assessed by the different standards.

In this study, 9 different feature extraction methods were investigated, under the condition of high throughput scanning prototype. A number of 200 bone marrow cells including 67 clinically meaningful chromosomes were first acquired. Then these cell images were processed and the feature extraction methods were applied for each acquired image. After that, the images were classified into analyzable and unanalyzable groups, using each feature extraction method. The classification performance of each feature was assessed by the ROC curve. The result shows that extracting the number of labeled regions is suitable for the on-line CAD scheme. For the off-line CAD scheme, it is recommended to use the number of labeled regions, average region area, average region pixel value, and the deviation of the either region circularity or distance. As mentioned before, the image features are application specific; thus the selected features cannot be directly used for other CAD programs as the optimal selections. However, the reported feature evaluating method is general and can be applied to optimize the performance of other CAD schemes.

As an initial study, however, this investigation has several limitations. First, the performance of the classifiers was not assessed in this investigation. In the CAD scheme, feature extraction and machine learning algorithm are two relatively independent parts. The machine learning algorithm uses the extracted features to group the analyzable (positive) and unanalyzable (negative) cells. Similar to the features, the performance of the different classifiers are also application dependent. The performance of classifiers, such as decision tree [31], support vector machine [33], fuzzy ARTMAP [34], and native Bayesian classifier [32], should be assessed uniformly to select the optimal one for the off-line CAD schemes. Second, we did not discuss the overall performance difference between the high throughput and conventional scanners, although the superiority of the high throughput scanning microscopy has been initially proved in our previous investigations [6]. Moreover, only few studies are focused on this comparison. Thus a more comprehensive study is prepared, which may be able to improve the accuracy of the high throughput scanning systems in the future.

## Figures and Tables

**Table 1:** The estimated *P* value of the difference significance between the features.

	The number of labeled regions	Average region area	Average pixel value	STD of the region circularity	STD of the region distance
The number of labeled regions	1	0	0	0	0
Average region area	0	1	0.1873	0.1484	0.4652
Average pixel value	0	0.1873	1	0.6576	0.6230
STD of the region circularity	0	0.1484	0.6576	1	0.3284
STD of the region distance	0	0.4652	0.6230	0.3284	1

**Table 2:** The estimated correlation coefficient among different features.

	The number of labeled regions	Average region area	Average pixel value	STD of the region circularity	STD of the region distance
The number of labeled regions	1	0.3253	0.1567	0.2939	0.3467
Average region area	0.3253	1	−0.0151	−0.1524	−0.1038
Average pixel value	0.1567	−0.0151	1	0.3698	0.3334
STD of the region circularity	0.2939	−0.1524	0.3698	1	0.6058
STD of the region distance	0.3467	−0.1038	0.3334	0.6058	1
